# Upfront Chemotherapy Versus Immediate Surgery for Operable Pancreatic Cancer: An Umbrella Review of Meta-Analyses

**DOI:** 10.3390/cancers18091344

**Published:** 2026-04-23

**Authors:** Michele Ghidini, Giuseppe Ietto, Lorenzo Dottorini, Andrea Celotti, Annamaria De Giorgi, Gianpaolo Balzano, Francesca Senzani, Gianluca Tomasello, Fausto Petrelli

**Affiliations:** 1 Medical Oncology Division, ASST Sette Laghi, 21100 Varese, Italy; michele.ghidini@uninsubria.it (M.G.);; 2 Department of Medicine and Technological Innovation (DiMIT), University of Insubria, 21100 Varese, Italygianpaolo.balzano@uninsubria.it (G.B.); 3 General, Emergency and Transplant Surgery Department, ASST Sette Laghi, 21100 Varese, Italy; 4 Oncology Unit, ASST Bergamo Ovest, 24047 Treviglio, Italy; 5 Surgery Unit, ASST Cremona, 26100 Cremona, Italy; 6 Surgery Unit, ASST Bergamo Ovest, 24047 Treviglio, Italy; frasenzani@gmail.com; 7 Oncology Unit, ASST Crema, 26013 Crema, Italy

**Keywords:** neoadjuvant therapy, pancreatic cancer, resectable

## Abstract

**Background**: Neoadjuvant therapy (NAT) is increasingly investigated in operable pancreatic ductal adenocarcinoma (PDAC), yet its role in strictly resectable disease remains controversial. Randomized trials have been conducted both in borderline resectable and resectable PDAC and have demonstrated survival advantages, while evidence in strictly resectable tumors remains poor. We conducted an umbrella review of systematic reviews and meta-analyses (SRMAs) to comprehensively evaluate the highest level of available evidence on NAT versus upfront surgery in operable PDAC. **Methods**: We performed an umbrella review of completed SRMAs assessing neoadjuvant chemotherapy (NAC) and/or chemoradiotherapy (NACRT) in resectable and borderline resectable PDAC. MEDLINE/PubMed, Embase, and Cochrane Library were searched from inception through November 2025. Eligible SRMAs reported at least one clinical outcome, including overall survival (OS), disease-free/event-free survival (DFS/EFS), resection rate, R0 resection, nodal status, or perioperative outcomes. Methodological quality was appraised using AMSTAR-2 and ROBIS tools. Overlap among SRMAs was quantified using the Corrected Covered Area (CCA), and RCT-only evidence was prioritized for causal inference. Evidence credibility was graded using an Ioannidis-style classification framework. **Results**: Thirty-four SRMAs published between 2010 and 2025 were included. In strictly resectable PDAC, RCT-only meta-analyses showed no definitive OS benefit for NAT compared with upfront surgery (pooled HR approximately 0.85, 95% CI 0.68–1.05), although a significant improvement in EFS was observed (HR approximately 0.77, 95% CI 0.65–0.90). Trial sequential analyses suggested insufficient information size for conclusive OS benefit in resectable disease. Conversely, in pooled resectable and borderline resectable populations, NAT significantly improved OS (HR approximately 0.66, 95% CI 0.52–0.85), with subgroup analyses indicating that the survival advantage was primarily driven by borderline resectable tumors. NAT consistently increased R0 resection and node-negative (pN0) rates and reduced non-curative explorations. However, neoadjuvant strategies were associated with treatment-related attrition and, in some analyses, lower overall resection rates. Comparative evidence suggested improved pathological outcomes with chemoradiotherapy versus chemotherapy alone, without a consistent survival advantage. **Conclusions**: Current high-level evidence supports NAT as the preferred strategy for borderline resectable PDAC, demonstrating consistent survival and pathological benefits. In strictly resectable disease, NAT improves disease-control endpoints and pathological surrogates, but a definitive OS advantage has not been consistently demonstrated in RCT-only syntheses. This should not be interpreted as evidence of equivalence between NAT and a surgery-first strategy, given the heterogeneity, limited power, and therapeutic-era effects of the available literature. Treatment decisions in resectable PDAC should therefore be individualized, balancing potential oncologic benefits against attrition risk. Future adequately powered randomized trials employing contemporary multi-agent regimens are needed to clarify the survival impact of NAT in strictly resectable disease.

## Simple Summary

Pancreatic ductal adenocarcinoma is an aggressive malignancy for which the optimal management of operable disease remains debated. While neoadjuvant therapy is standard for borderline resectable tumors, its role in strictly resectable disease is uncertain. We conducted an umbrella review of 34 systematic reviews and meta-analyses to synthesize the highest level of evidence comparing neoadjuvant therapy with upfront surgery. In borderline resectable cancer, neoadjuvant therapy consistently improved survival and surgical outcomes. In strictly resectable disease, randomized-trial evidence showed better disease-control outcomes and improved pathological features, but no definitive overall survival advantage. These findings should not be interpreted as equivalence between strategies, as available trials remain underpowered and heterogeneous. Treatment decisions for resectable pancreatic cancer should therefore be individualized, balancing potential oncologic benefits with the risk of treatment-related attrition. Further adequately powered randomized trials using modern combination regimens are needed to clarify the survival impact of neoadjuvant therapy in strictly resectable disease.

## 1. Introduction

Upfront surgical resection followed by adjuvant chemotherapy remains a major indication for the treatment of resectable pancreatic ductal adenocarcinoma (PDAC) [1] and it is endorsed by the National Comprehensive Cancer Network (NCCN) [2], American Society of Clinical Oncology (ASCO) [3] and European Society of Medical Oncology (ESMO) guidelines [4]. However, approximately 65% of patients do not commence adjuvant therapy due to the detection of metastases during or after surgery and because of postoperative morbidity or deterioration of clinical conditions following surgical intervention [5].

Neoadjuvant chemotherapy (NAT) offers many advantages. Firstly, it allows eradication of occult micrometastatic disease. Secondly, the administration of preoperative treatment selects non-progressor patients who are most likely to benefit from surgery and, thirdly, it permits higher rates of radical resection (R0) within tumor downsizing. In borderline resectable PDAC, NAT improved R0 resection rates and improved survival so that guidelines endorse this strategy as the preferred one in this setting [6].

Differently, the role of NAT in resectable disease remains a topic of debate. NAT was explored in randomized phase 2 trials with promising results in both resectable and borderline resectable populations. However, the anatomical resectability classification has not had prognostic validation and it is hardly reproducible [5]. The phase III PREOPANC-1 study enrolled both resectable or borderline resectable PDAC and showed an improved overall survival (OS) with neoadjuvant gemcitabine-based chemoradiotherapy followed by surgery and adjuvant gemcitabine with respect to upfront surgery [7]. The following PREOPANC-2 trial aimed to evaluate whether neoadjuvant FOLFIRINOX improved OS compared with neoadjuvant gemcitabine-based chemoradiotherapy followed by adjuvant gemcitabine in the same setting of patients. The trial did not show a difference in survival outcomes between the two different regimens, with FOLFIRINOX becoming the preferred option for higher feasibility [8]. Recently, the phase 3 CASSANDRA trial enrolled resectable and borderline resectable PDAC and compared a neoadjuvant modified FOLFIRINOX regimen (mFOLFIRINOX) with combination chemotherapy with cisplatin, nab-paclitaxel, gemcitabine and capecitabine (PAXG). PAXG prolonged the median event-free survival (EFS) compared with mFOLFIRINOX (16.0 months vs. 10.2 months, hazard ratio [HR] 0·63, *p* = 0.0018) and became a candidate new standard regimen for this setting [5].

Despite these recent results, which strongly support a neoadjuvant strategy both in resectable and borderline resectable PDAC, NAT is still underused and upfront resection remains a widespread option when disease is approachable with surgery. In our work, we performed an umbrella review of systematic reviews and meta-analyses (SRMAs) available in this setting of patients. Umbrella reviews systematically evaluate multiple meta-analyses on the same topic, provide a unique opportunity to synthesize the highest level of evidence, identify areas of consensus and controversy and assess the quality and credibility of available evidence using rigorous methodological frameworks.

## 2. Methods

This systematic review and Bayesian network meta-analysis were conducted and re-ported in accordance with the Preferred Reporting Items for Systematic Reviews and Meta-Analyses (PRISMA) 2020 statement (Supplemental Materials Tables S1–S4). The protocol was not prospectively registered in PROSPERO or any other systematic review registry.

### 2.1. Study Design and Framework

We conducted an umbrella review (UR) of systematic reviews and meta-analyses (SRMAs) evaluating neoadjuvant strategies in operable PDAC. The UR was designed and reported according to contemporary methodological guidance for umbrella reviews, including stepwise study selection, overlap management, methodological quality appraisal, and evidence-credibility grading.

### 2.2. Eligibility Criteria

We included completed SRMAs that quantitatively synthesized outcomes of NAT—including neoadjuvant chemotherapy (NAC) and/or neoadjuvant chemoradiotherapy (NACRT)—in resectable PDAC. Eligible SRMAs had to report at least one of the following outcomes: overall survival (OS), disease-free/event-free survival (DFS/EFS), resection rate, R0 resection, nodal status (N0/pN0), perioperative morbidity or mortality.

We excluded narrative reviews, protocols without results, single trials, and SRMAs focused exclusively on metastatic disease.

### 2.3. Search Strategy and Study Identification

We searched MEDLINE/PubMed, Embase, and the Cochrane Library (CDSR and CENTRAL) for systematic reviews and meta-analyses evaluating neoadjuvant or preoperative treatment strategies in operable pancreatic ductal adenocarcinoma. The search window spanned from inception through 30 November 2025, and no language restrictions were applied at the search stage. The search strategy was: ((“Pancreatic Neoplasms”[Mesh] OR pancreas cancer*[tiab] OR pancreatic cancer*[tiab] OR pancreatic neoplasm*[tiab] OR pancreatic ductal adenocarcinoma[tiab] OR PDAC[tiab]) AND (resect*[tiab] OR operab*[tiab] OR “borderline resectable”[tiab] OR localized[tiab] OR nonmetastatic[tiab] OR “non-metastatic”[tiab]) AND (neoadjuvant[tiab] OR preoperativ*[tiab] OR “induction therapy”[tiab] OR “total neoadjuvant”[tiab] OR chemotherap*[tiab] OR chemoradi*[tiab] OR radiotherap*[tiab] OR “radiation therapy”[tiab]) AND (surgery[tiab] OR resection[tiab] OR “upfront surgery”[tiab] OR “surgery first”[tiab] OR “immediate surgery”[tiab])) AND (“systematic review”[Publication Type] OR “meta-analysis”[Publication Type] OR (systematic[tiab] AND review[tiab]) OR meta-analys*[tiab]). Records were restricted to systematic reviews and meta-analyses using database-specific filters and publication types. All retrieved citations were exported to a reference manager and deduplicated using identifiers and title matching.

**Figure 1:**
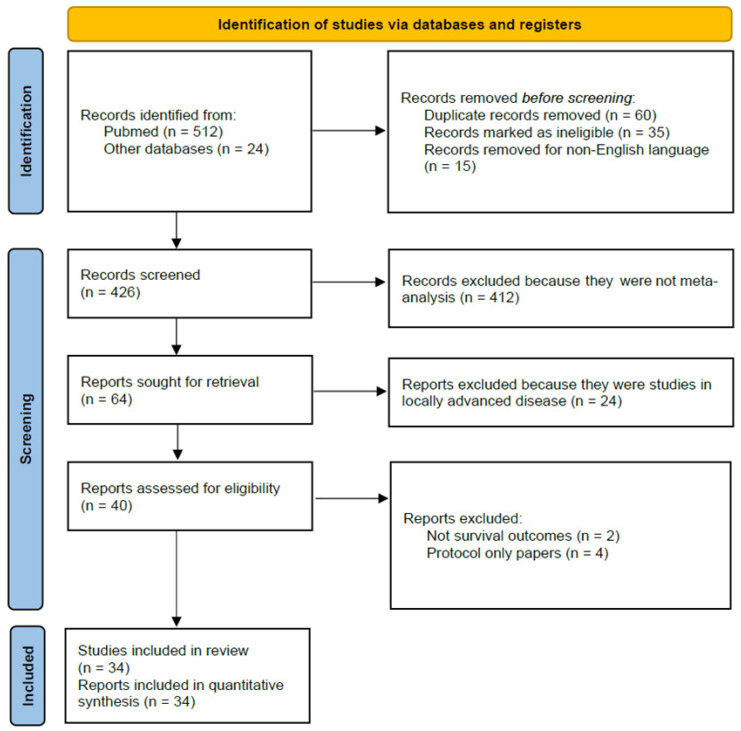
PRISMA flow diagram of included studies.

Two reviewers (FP and MG) independently screened titles and abstracts to identify potentially eligible evidence syntheses. Full texts were then assessed in duplicate against prespecified eligibility criteria, retaining completed systematic reviews and meta-analyses that quantitatively synthesized outcomes of NAT, including NAC and/or NACRT, in resectable PDAC and reported at least one clinical endpoint of interest such as OS, disease-free survival (DFS) or EFS, resection rate, R0 resection, nodal status, or perioperative morbidity and mortality. Narrative reviews, protocols without results, single trials, and syntheses limited to metastatic disease were excluded. To ensure completeness, reference lists of included meta-analyses were hand-searched and forward citation tracking was performed for the most recent and methodologically rigorous reviews. The PRISMA flow diagram of included studies is described in Figure 1.

### 2.4. Data Extraction

From each SRMA we extracted: population and resectability definition; intervention/comparator; number and type of primary studies; pooled effect estimates (HR, RR, OR, or pooled medians as reported); heterogeneity metrics (I^2^); publication-bias assessments; and key safety outcomes. When multiple effect measures were reported, intention-to-treat (ITT) estimates were prioritized.

### 2.5. Methodological Quality and Risk of Bias

Methodological quality was assessed using AMSTAR-2, with attention to critical domains (protocol registration, comprehensive search, duplicate processes, risk-of-bias assessment, appropriate synthesis, publication-bias assessment). Overall risk of bias was assessed using ROBIS. An integrated UR quality grade was assigned (High, Medium, Low) based on concordance between AMSTAR-2 and ROBIS (Table 1).

Overlap cluster: a label grouping SRMAs that address the same clinical question and draw from substantially overlapping primary study pools. Within each cluster, one SRMA is designated as Index (recommended primary reference); the others serve for triangulation and sensitivity analysis. Clusters are coded by letter (A = NAT vs. upfront surgery; B = specific treatment strategy; C = total neoadjuvant therapy; D = early/broad single-arm SRMAs) and number (reflecting subgroup by resectability scope and study design.

### 2.6. Overlap Assessment and Management

Because multiple SRMAs addressed identical PICOs, overlap of primary studies was quantified using the Corrected Covered Area (CCA). We quantified overlap using the Corrected Covered Area (CCA), calculated as: CCA = (C − S)/[S × (r − 1)] where C = total number of study inclusions across SRMAs, S = number of unique primary studies, and r = number of SRMAs. CCA was classified as slight (≤5%), moderate (6–10%), high (11–15%), or very high (>15%).

**Figure 2:**
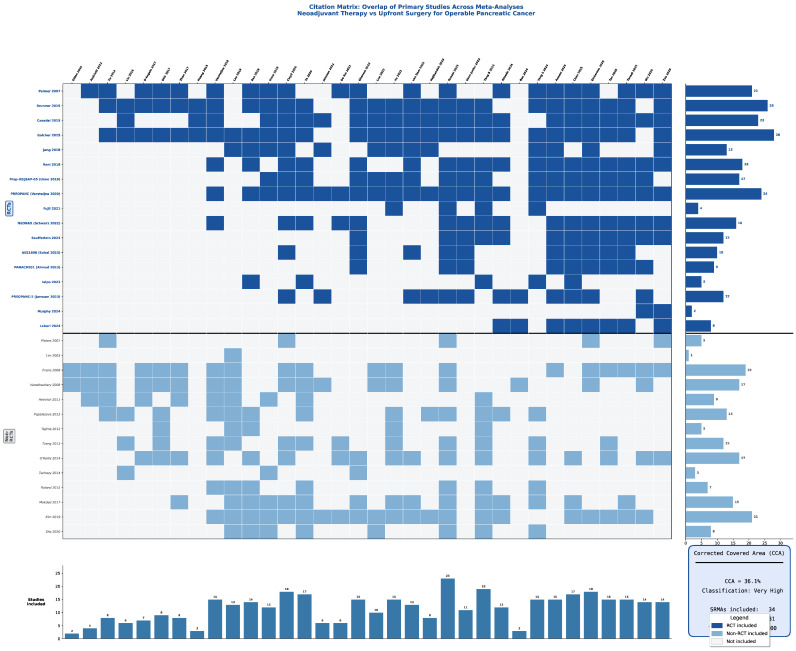
Citation matrix: overlap of primary studies across meta-analyses.

For clusters with high/very high overlap (notably RCT-only NAT vs. upfront surgery), a single-index SRMA was selected based on methodological quality, recency, and analytic rigor, with remaining SRMAs used for triangulation and sensitivity (Figure 2).

### 2.7. Evidence Synthesis and Credibility Grading

Findings were synthesized qualitatively and quantitatively at the outcome level. RCT-only SRMAs were prioritized for causal inference; mixed-design SRMAs were considered supportive/contextual. Evidence credibility was graded using an Ioannidis-style hierarchy (Class I–IV; non-significant), applying conservative criteria given incomplete reporting of prediction intervals and small-study effects in many SRMAs.

### 2.8. Statistical Analysis

As this is an umbrella review, no de novo SRMA of primary study data was performed. The unit of analysis included each SRMA, and the principal analytical approach was qualitative and semi-quantitative synthesis at the outcome level. For each included SRMA, we extracted the reported pooled effect estimates (hazard ratios [HR], risk ratios [RR], or odds ratios [OR]) with their 95% confidence intervals (CIs) and heterogeneity statistics (I^2^). When multiple analyses were reported within a single SRMA (e.g., intention-to-treat and per-protocol), the intention-to-treat estimate was prioritized. Methodological quality of each SRMA was assessed using AMSTAR-2 with attention to all 16 domains including the 7 critical domains. Risk of bias at the SRMA level was evaluated using the ROBIS tool. Evidence credibility was graded using the Ioannidis-style classification framework, incorporating statistical significance at *p* < 0.05, sample size (>1000 cases), low heterogeneity (I^2^ < 50%), absence of small-study effects, and absence of excess significance bias. Evidence was classified as Class I (convincing), Class II (highly suggestive), Class III (suggestive), Class IV (weak), or non-significant. CCA calculations and citation matrix generation were performed using Python 3.11 (Python Software Foundation) with NumPy and Matplotlib libraries (https://www.python.org/; last accessed 12 March 2026). A biostatistician (FP) reviewed the analytical framework, CCA calculations, evidence credibility grading, and all statistical outputs prior to manuscript submission, and is listed in the acknowledgments section.

## 3. Results

### 3.1. Study Set and Design Features

This umbrella review synthesized the prespecified set of 34 systematic reviews/meta-analyses on operable pancreatic cancer (resectable and/or borderline resectable PDAC), covering the period 2010–2025 and including RCT-only meta-analyses, reconstructed/IPD meta-analyses, and mixed-design syntheses [6,9,10,11,12,13,14,15,16,17,18,19,20,21,22,23,24,25,26,27,28,29,30,31,32,33,34,35,36,37,38,39,40,41] (Table 1, Table 2 and Table 3: Supplementary Tables S1–S3). Early evidence was dominated by single-arm phase I–II and cohort/case-series data (e.g., Gillen 2010 [40], 111 studies; n = 4394). The number of total unique primary studies identified across all SRMAs was 31 (17 RCTs + 14 non-randomized comparative studies), while the total study inclusions across SRMAs was 400 (reflecting repeated inclusion of the same studies).

### 3.2. Overlap Across Meta-Analyses

Substantial overlap was present among meta-analyses addressing the same PICO, particularly RCT-only NAT vs. upfront surgery in resectable PDAC and RCT-only NAT vs. upfront surgery in pooled resectable/borderline cohorts, due to repeated inclusion of the same core randomized trials. Consequently, outcome-level synthesis prioritized the most methodologically robust and/or most recent RCT-focused meta-analyses, with other overlapping meta-analyses used for triangulation and to explain discordance driven by differences in trial inclusion, endpoint definition (especially R0), and analytic choices (ITT vs. reconstructed survival) (Figure 2).

### 3.3. Methodological Quality and Risk of Bias (SRMA-Level)

Recent RCT-focused SRMAs generally reported PRISMA-concordant methods and formal trial risk-of-bias appraisal (e.g., RoB-2) and, in some cases, additional robustness methods such as reconstructed survival and trial sequential analysis (TSA). In contrast, earlier large syntheses (e.g., Gillen 2010) were comprehensive but intrinsically limited by indirectness and heterogeneity, given inclusion of predominantly nonrandomized studies and the lack of direct controlled comparisons; Gillen explicitly notes these limitations and the assumptions required for survival estimation [40].

### 3.4. Primary Outcomes

#### Overall Survival (OS)

- Resectable PDAC (RCT-only evidence)

Across contemporary RCT-only syntheses in strictly resectable disease, OS benefit was not consistently demonstrated. In the largest updated RCT-only meta-analysis with reconstructed survival and TSA (9 RCTs; 604 NAT vs. 527 upfront surgery), NAT showed a non-significant trend toward improved OS (HR 0.85, 95% CI 0.68–1.05), with TSA indicating OS remained inconclusive due to insufficient accumulated information size. This “no definitive OS advantage” conclusion is concordant with other resectable-only RCT syntheses cited within the corpus, where OS estimates generally crossed unity despite favorable point estimates.

- Resectable + borderline resectable (RCT-only evidence)

When resectable and borderline resectable tumors were pooled in RCT-only evidence (7 trials; n = 938), NAT improved OS (HR 0.66, 95% CI 0.52–0.85; I^2^ = 46%). Importantly, this meta-analysis reported that all included trials used gemcitabine-based chemo(radio)therapy, highlighting regimen-era limitations.

The same RCT-only synthesis reported that the OS benefit was more pronounced in borderline resectable disease than in resectable disease, supporting effect heterogeneity by resectability status.

- Disease-control endpoints (EFS/DFS)

In the largest updated resectable-only RCT meta-analysis (9 RCTs), NAT yielded a significant EFS benefit (HR 0.77, 95% CI 0.65–0.90) with TSA supporting that the EFS effect was definitive, while OS remained inconclusive.

Other RCT-focused syntheses in the corpus also reported DFS/EFS advantages variably depending on included trials and regimen categories.

### 3.5. Secondary Outcomes

#### 3.5.1. R0 Resection and Nodal Downstaging

- Resectable PDAC (RCT-only)

For resectable PDAC, R0 resection improvements were directionally consistent across RCT-focused evidence but varied in statistical certainty. In the largest RCT-only updated synthesis, R0 favored NAT numerically but did not reach conventional statistical significance (RR 1.13, 95% CI 1.00–1.27), and TSA suggested R0 remained inconclusive.

In contrast, other resectable-only RCT meta-analyses reported significant improvements in R0 and pN0, reflecting differences in included trial sets and margin definitions.

Notably, the updated RCT-only synthesis reported that NAT reduced noncurative surgeries (exploration RR 0.90, 95% CI 0.87–0.94) and increased node-negative histology (pN0 RR 1.73, 95% CI 1.31–2.28).

- Resectable + borderline resectable (RCT-only)

In the RCT-only R/BR synthesis, R0 and N0 were explicitly reported as surrogate outcomes and therefore judged as indirectly relevant in GRADE terminology, but nonetheless favored NAT (R0 more common after NAT in the synthesis; N0 similarly improved).

- Neoadjuvant CRT vs. immediate surgery (RCT-only)

In the RCT-only CRT-vs-surgery synthesis, neoadjuvant CRT increased R0 overall (RR 1.55; *p* = 0.004) and reduced overall resection rate (RR 0.83; *p* = 0.008). Stratification suggested that the improvement in R0 and OS was driven primarily by BRPC rather than RPC.

#### 3.5.2. Resection Rate and Attrition

Across RCT-only evidence, resection rates showed either no clear difference or a modest reduction with NAT depending on the comparison and disease mix. In the RCT-only R/BR analysis, the pooled resection rate was not statistically different in the main analysis (RR 0.94; 95% CI 0.89–1.01; *p* = 0.08), while the CRT-vs-surgery RCT analysis demonstrated lower overall resection rates with neoadjuvant CRT (RR 0.83; *p* = 0.008), consistent with attrition/progression during preoperative therapy.

- Regimen comparison: chemoradiotherapy vs. chemotherapy (after NAT; mixed designs)

In a comparative meta-analysis assessing CRT vs. chemotherapy (11 studies; n = 3571 resected after NAT; baseline stages included resectable, borderline resectable, and locally advanced), CRT was associated with higher pCR (OR 3.58) and higher R0 (OR 1.49), but no difference in 3-year OS (OR 1.07, 95% CI 0.84–1.36).

#### 3.5.3. Safety Outcomes

In the early comprehensive synthesis, grade 3/4 toxicity of neoadjuvant therapy was substantial (approximately 29% overall) with high heterogeneity; morbidity and mortality after resection were higher in initially non-resectable cohorts than in initially resectable cohorts, emphasizing case-mix and surgical complexity effects. In modern RCT-only resectable PDAC evidence, postoperative morbidity and mortality were reported as broadly comparable between NAT and upfront surgery, with systemic treatment-related adverse events varying across regimens and trials.

## 4. Discussion

This umbrella review synthesized evidence from 34 systematic reviews and meta-analyses published between 2010 and 2025, providing one of the most comprehensive evaluations to date of neoadjuvant therapy versus upfront surgery for operable PDAC. The findings indicate that the benefit of NAT is strongly contingent on resectability status, regimen composition, and the endpoints examined, and they highlight trade-offs that should shape clinical decision-making and future trial design (Supplementary Table S4).

A consistent and clinically meaningful theme is effect heterogeneity by resectability category. In strictly resectable disease, contemporary RCT-only evidence does not demonstrate a definitive OS advantage for neoadjuvant therapy. The most methodologically rigorous synthesis reported an OS hazard ratio of 0.85 (95% CI 0.68–1.05) and trial sequential analysis suggested that the information size remains insufficient to draw firm conclusions. This challenges the premise that NAT should be uniformly adopted for all resectable pancreatic cancers and supports upfront surgery followed by adjuvant therapy as an evidence-based option. However, the favorable outcomes historically associated with surgery-first strategies should be interpreted in light of the selection embedded in adjuvant trials, which predominantly enroll patients who have already undergone successful resection, recovered adequately, and remained fit enough to receive postoperative systemic therapy; as such, they may overestimate the real-world population effectiveness of a surgery-first pathway. At the same time, the same RCT-focused evidence shows a consistent EFS benefit (HR 0.77, 95% CI 0.65–0.90) with conclusive trial sequential analysis, suggesting that neoadjuvant therapy can delay recurrence even when an OS signal is not established. This dissociation between disease-control and survival endpoints may reflect competing risks, limited trial power, attrition effects, or the possibility that improved local-pathologic endpoints do not fully capture the dominant systemic biology of resectable PDAC [35].

In contrast, when resectable and borderline resectable tumors are pooled, NAT is associated with a clear OS advantage (HR 0.66, 95% CI 0.52–0.85), and subgroup analyses consistently indicate that this benefit is driven primarily by borderline resectable disease. In borderline resectable pancreatic cancer specifically, NACRT was associated with substantial survival improvements compared with immediate surgery (mean difference 6.64 years; *p* = 0.004), whereas the effect in resectable disease was minimal and non-significant (mean difference 0.94 years; *p* = 0.57). These findings have direct clinical implications: patient selection based on anatomic resectability criteria should be central to treatment planning, and the strongest evidence-based indication for a neoadjuvant approach is borderline resectable disease. Mechanistically, this resectability-dependent benefit is biologically plausible. Borderline tumors have a higher likelihood of occult systemic disease and a greater risk of margin-positive resection; both risks may be mitigated by early systemic therapy and local downstaging, improving the probability that surgery delivers meaningful oncologic value [20,39].

Secondary endpoints provide additional insight into mechanisms and trade-offs. Across multiple meta-analyses, neoadjuvant therapy consistently increased rates of margin-negative (R0) resection and node-negative histology (pN0). In the most robust RCT-only synthesis, neoadjuvant therapy increased pN0 rates by 73% (RR 1.73, 95% CI 1.31–2.28) and reduced non-curative explorations (RR 0.90, 95% CI 0.87–0.94). These findings suggest both biologic efficacy (tumor regression and nodal sterilization) and enhanced selection of patients with favorable tumor biology who are most likely to benefit from resection. They also underscore that, in resectable PDAC, pathway-related endpoints remain clinically meaningful even when OS differences are not definitive, including the opportunity to deliver systemic therapy upfront, identify early progressors before surgery, and improve the overall oncologic quality of resection. However, the incomplete translation of these surrogate gains into consistent OS benefits in resectable disease raises two considerations. First, margin and nodal status may be imperfect surrogates for survival in a disease where early systemic dissemination is common. Second, countervailing factors—treatment-related attrition, delays to surgery, or enrichment of resistant clones—may offset improvements in operative–pathologic outcomes at the population level [6].

Attrition is a pivotal issue. Several meta-analyses observed lower overall resection rates with neoadjuvant strategies, particularly NACRT (e.g., RR 0.83; *p* = 0.008), reflecting progression, toxicity, or clinical deterioration during preoperative therapy. This represents a fundamental trade-off: neoadjuvant therapy may improve outcomes among those who complete therapy and proceed to surgery, but it may also prevent some patients from reaching potentially curative resection. Conversely, surgery-first pathways are also vulnerable to attrition, because a proportion of resected patients never receive planned adjuvant chemotherapy owing to postoperative morbidity, delayed recovery, or early relapse. Because this attrition directly affects intention-to-treat outcomes, ITT estimates should be prioritized when counselling patients and when designing trials. The magnitude and consequences of attrition are likely context-dependent, influenced by baseline performance status, comorbidity burden, treatment intensity, restaging frequency, and institutional experience. Minimizing avoidable attrition—through careful selection, proactive supportive care, and timely transitions to surgery when necessary—is therefore an essential component of implementing NAT in practice [20,33].

Evidence regarding the addition of radiotherapy remains mixed and outcome specific. Comparative syntheses indicate that NACRT improves pathologic outcomes compared with chemotherapy alone (higher R0; OR 1.49, and higher pCR; OR 3.58), but does not improve 3-year OS (OR 1.07, 95% CI 0.84–1.36). These findings align with ongoing debates about radiotherapy’s role in PDAC: it may enhance local control and margin negativity, but survival benefits are uncertain, and radiotherapy can contribute to delays and attrition. As a result, radiotherapy may be best viewed as a selective component—particularly for borderline resectable disease, persistent vascular interface after induction chemotherapy, or settings where local control is prioritized—pending further randomized evidence with modern systemic backbones [11].

Recurrence pattern data were inconsistently reported across the included SRMAs, precluding formal synthesis. However, individual RCTs provide informative signals. In the PREOPANC-1 trial, neoadjuvant chemoradiotherapy was associated with a significantly lower locoregional recurrence rate compared with upfront surgery (30% vs. 47%), while distant recurrence rates were similar between arms (approximately 70% in both groups). In the Prep-02/JSAP-05 trial, neoadjuvant gemcitabine plus S-1 reduced hepatic recurrence compared with upfront surgery (30% vs. 47.5%), suggesting a systemic effect of preoperative therapy.

The NEONAX trial reported that despite improved R0 and pN0 rates with perioperative nab-paclitaxel/gemcitabine, the predominant pattern of failure remained distant metastasis (liver and peritoneum), highlighting the systemic nature of pancreatic cancer even in the resectable setting. Similarly, the Alliance A021806 trial observed that the first recurrence was distant in the majority of patients in both arms.

These data collectively suggest that neoadjuvant therapy may improve local control (consistent with the observed improvements in R0 and pN0 rates across our umbrella review), but the dominant failure pattern remains systemic—which may partly explain why pathological surrogates do not fully translate into consistent OS benefits in resectable disease. Future meta-analyses specifically designed to synthesize recurrence-pattern data, ideally using individual patient data, would be valuable to address this gap. Site-specific recurrence data (local vs. distant) were not systematically reported across SRMAs and could not be formally synthesized; available trial-level data suggest improved local control with NAT but persistent systemic failure.

A critical limitation shared by virtually all meta-analyses in this umbrella review is the predominance of gemcitabine-based regimens in the underlying RCT evidence. Of the core randomized trials that inform current syntheses, the majority—including PREOPANC-1, Prep-02/JSAP-05, and most earlier phase II trials—employed gemcitabine monotherapy or gemcitabine-based chemoradiotherapy as the neoadjuvant backbone. This raises the fundamental question of whether the observed effect sizes, particularly the non-significant OS trend in resectable disease (HR approximately 0.85), reflect a true ceiling of NAT efficacy or simply the limitations of less active systemic regimens.

Several lines of evidence suggest that results with contemporary multi-agent chemotherapy may differ substantially. In the metastatic setting, FOLFIRINOX demonstrated a median OS of 11.1 months versus 6.8 months for gemcitabine (PRODIGE-4/ACCORD-11), and gemcitabine plus nab-paclitaxel achieved 8.5 months versus 6.7 months (MPACT). These regimens are now standard for fit patients with advanced disease, and their superior systemic activity is expected to translate into more effective eradication of micrometastatic disease in the neoadjuvant setting [16,39,40].

The recently reported CASSANDRA trial provides the first phase III evidence directly comparing two modern multi-agent neoadjuvant regimens—PAXG (cisplatin, nab-paclitaxel, capecitabine, and gemcitabine) versus modified FOLFIRINOX—in resectable and borderline resectable PDAC. PAXG achieved a significant EFS advantage (median 16.0 vs. 10.2 months; HR 0.63, 95% CI 0.47–0.84; *p* = 0.0018), along with higher rates of CA 19-9 response (72% vs. 55%), pathological complete response (16% vs. 4%), and node-negative resections (65% vs. 48%). Importantly, the trial found no independent prognostic role for anatomical resectability classification, suggesting that biological tumor features, rather than radiologic vascular relationships, may be more relevant for treatment stratification. Although overall survival data from CASSANDRA remain immature, these results strongly support that more active neoadjuvant regimens may overcome the modest effect sizes observed in gemcitabine-era trials.

In practical terms, three implications follow. First, the pooled HR estimates for OS reported across SRMAs in this umbrella review should be interpreted as conservative lower bounds of NAT efficacy, as they largely reflect gemcitabine-era data. Second, the significant EFS benefit (HR 0.77) observed even with older regimens suggests that more potent systemic therapy could plausibly shift the OS curve beyond the current threshold of statistical significance. Third, and perhaps most importantly, the ongoing evolution toward modern regimens reinforces the need for new, adequately powered RCTs comparing contemporary NAT (such as FOLFIRINOX, PAXG, or emerging combinations) against upfront surgery with modern adjuvant therapy in strictly resectable disease. Until such trials mature, the present umbrella review represents the best available synthesis of existing evidence, while acknowledging that its conclusions are necessarily regimen-era dependent [5].

Clinical implications follow a risk-stratified logic. For borderline resectable disease, neoadjuvant therapy should be considered the preferred approach given consistent survival benefit, improved R0 rates, and enhanced selection [42,43]. For strictly resectable disease, the choice between neoadjuvant therapy and upfront surgery should be individualized. The absence of definitive OS improvement in RCT-only evidence supports upfront surgery with adjuvant therapy as an appropriate standard pathway. Nevertheless, neoadjuvant therapy may be reasonable for “biologically high-risk” resectable patients (e.g., markedly elevated CA 19-9, large primary tumors, suspicious nodes, or other high-risk imaging or clinical features), where early systemic control and improved selection may be particularly valuable. Shared decision-making should explicitly address the trade-off between potential improvements in disease-control and pathologic endpoints versus the risk of attrition and delayed resection [6,9,15,20].

Methodologically, this umbrella review has several strengths: a comprehensive synthesis of SRMAs, explicit prioritization of RCT-only evidence for causal inference, and incorporation of structured appraisal approaches (AMSTAR-2/ROBIS) and overlap-aware evidence handling. However, limitations are unavoidable. Substantial overlap across SRMAs means that the apparent breadth of evidence often reflects repeated synthesis of a relatively small core trial set. Many SRMAs do not report prediction intervals or formal assessments of small-study effects and excess significance, limiting the ability to assign the highest credibility grades. Heterogeneity in resectability definitions and in R0 margin thresholds (0 mm vs. 1 mm) introduces additional interpretive complexity. Most importantly, regimen era effects limit applicability to modern neoadjuvant strategies [12,13].

Future research priorities are clear. Randomized trials comparing modern multi-agent neoadjuvant chemotherapy regimens against upfront surgery in strictly resectable disease are needed, including those that are adequately powered for OS and incorporate quality-of-life and patient-reported outcomes. In this direction, trials should also define optimal duration and sequencing, including the role of total neoadjuvant therapy, and should clarify when radiotherapy adds value (and for whom), ideally incorporating modern techniques such as SBRT where appropriate. Biomarker development is equally critical to refine patient selection beyond anatomy, using molecular profiling, ctDNA, radiomics, and dynamic CA 19-9 response to identify patients most likely to benefit and those at a high risk of progression/attrition. Finally, implementation and health-systems research should address variability in real-world adoption, the impact of center volume and multidisciplinary pathways, and cost-effectiveness, particularly given the logistical burden of neoadjuvant therapy [14,20,21,27].

## 5. Conclusions

The current evidence supports NAT as the preferred strategy for borderline resectable PDAC and as a selective option for resectable disease, where benefits in EFS and pathologic endpoints are evident but definitive OS improvement has not been consistently demonstrated in RCT-only syntheses. Importantly, the absence of definitive evidence for superiority of NAT in strictly resectable disease should not be interpreted as proof of equivalence with a surgery-first strategy, given the heterogeneity of the literature, limited statistical power, and underrepresentation of modern regimens. Until modern regimen-based randomized trials mature, clinicians should apply a resectability- and biology-informed approach, balancing disease-control gains against attrition risk within experienced multidisciplinary programs.

## Figures and Tables

**Table 1:** Methodological quality and overlap management.

**SRMA (Year; Journal)**	**Main PICO Node**	**Operable Scope**	**Design**	**RoB Tool Reported**	**Pub-Bias Methods Reported**	**AMSTAR-2**	**ROBIS**	**Integrated Grade**	**Overlap Cluster**	Index SRMA?
Tan HL (2025; *Surgery* [6]	NAT vs. upfront surgery	Resectable	SR/MA RCTs + TSA + survival reconstruction	RoB-2	Funnel + Egger	Moderate	Low	High	A1 (RPC, RCT SRMAs)	Yes
Chan AHY (2025; *Ann Surg Oncol*) [9]	NAT vs. upfront surgery	Resectable	SR/MA RCTs	RoB-2	Egger/funnel	Moderate	Low	High	A1	No
Tanadi C (2025; *Ann Gastroenterol*) [10]	NAT vs. upfront surgery	Resectable	SR/MA RCTs	RoB-2 + GRADE	Funnel if >10	Moderate	Unclear	Medium	A1	No
Zuo H (2025; *Medicine*) [11]	NAT vs. upfront surgery	Resectable	SR/MA (RCT focus)	Reported (trial RoB)	Not clearly verifiable	Low–Moderate	Unclear	Medium	A1	No
Aliseda D (2024; *BJS Open*) [12]	NAT vs. upfront surgery	Resectable	Reconstructed patient-level MA of RCTs	RoB-2	Funnel/Egger	High–Moderate	Low	High	A1	No
Ghanem I (2022; *ESMO Open*) [13]	NAC ± RT vs. surgery	Resectable	SR/MA RCTs	RoB-2/Cochrane	Funnel	Moderate	Low	High	A1	No
Uson Junior PLS (2023; *ESMO Open*) [14]	NAT vs. surgery	Resectable	SR/MA RCTs	RoB (reported)	Not clearly verifiable	Low	Unclear	Low–Medium	A1	No
van Dam JL (2022; *Eur J Cancer*) [15]	NAT vs. upfront surgery	Resectable + BRPC	SR/MA RCTs + GRADE	Cochrane RoB	Not central; RCT-only	High–Moderate	Low	High	A2 (R/BR, RCT SRMAs)	Yes
Cloyd JM (2020; *J Clin Med*) [16]	NAT vs. upfront surgery	Resectable + BRPC	SR/MA RCTs	RoB tool reported	Not clearly verifiable	Moderate	Unclear–Low	Medium–High	A2	No
Dickerson LD (2025; *BJS Open*) [17]	NAT vs. upfront surgery	Resectable + BRPC	SR/MA (trials)	RoB tool reported	Not central	Moderate	Unclear	Medium	A2	No
Annesi CA (2025; *J Surg Res*) [18]	NAT vs. upfront surgery	Resectable + BRPC	SR/MA RCTs	RoB tool reported	Not clearly verifiable	Moderate	Unclear	Medium	A2	No
Hajibandeh S (2023; *Ann Hepatobiliary Pancreat Surg*) [19]	NCRT vs. surgery-first	Resectable + BRPC	SR/MA + TSA	Cochrane RoB	Funnel if ≥10	Moderate	Unclear	Medium	B1 (CRT vs. surgery-first)	Yes
Wu HY (2025; *IJROBP*) [20]	NAC vs. NAC-CRT (and CRT role)	Resectable + BRPC	SR/MA (prospective; includes RCTs)	Reported	Reported	Moderate	Unclear	Medium	B4 (CRT/RT strategy SRMAs)	Yes
Janssen QP (2021; *Ann Surg Oncol*) [21]	FOLFIRINOX ± RT	Resectable + BRPC	SR/MA (mostly nonrandomized)	CASP/quality tool	Not central	Low–Moderate	High	Low	B3 (RT add-on after NAC)	Yes
Bao QR (2024; *Pancreatology*) [22]	CT vs. CRT (pathologic response)	Mixed/unclear	SR/MA (mostly nonrandomized)	Quality tool	Reported	Moderate	High	Medium	B2 (CT vs. CRT)	Yes
Luo W (2022; *Front Oncol*) [23]	NCRT vs. surgery-first	Resectable/mixed	SR/MA (mixed)	Quality tool	Not clearly verifiable	Low–Moderate	High–Unclear	Low	B4	No
Liu W (2016; *Medicine*) [24]	NCRT vs. surgery	Resectable	SR/MA (mixed; older era)	NOS/Cochrane	Not clear	Low	High	Low	B4	No
Xu CP (2014; *J Cancer Res Clin Oncol*) [25]	CRT vs. non-CRT	Resectable	SR/MA (mixed; older era)	Quality tool (older)	Not clear	Low	High	Low	B4	No
Huang L (2018; *Am Surg*) [26]	NCRT→surgery vs. surgery	Resectable	SR/MA	Not clear	Not clear	Low	High–Unclear	Low	B4	No
De Simoni O (2022; *J Clin Med*) [27]	TNT	Mixed/unclear	SR/MA	NOS/quality tool	Not clear	Low–Moderate	High	Low	C1 (TNT)	Yes
Yang B (2023; *Clin Exp Med*) [28]	NAT vs. surgery	Resectable	SR/MA (mixed)	NOS/quality tool	Not clear	Low–Moderate	High	Low	A3 (RPC mixed SRMAs)	No
Yang SQ (2024; *Updates Surg*) [29]	NAT vs. surgery	Resectable + BRPC	SR/MA (mixed)	NOS + Cochrane	Not clear	Low–Moderate	High–Unclear	Low	A4 (R/BR mixed SRMAs)	No
Roesel R (2023; *Cancers*) [30]	NAT vs. surgery	Resectable	SR/MA (mixed)	Quality tool	Not clear	Low–Moderate	High–Unclear	Low	A3	No
Ye M (2020; *HPB*) [31]	Neoadj chemotherapy	Resectable	SR/MA (mixed)	NOS	Not clear	Low–Moderate	High	Low	A3	No
Pan L (2020; *WJSO*) [32]	NAT vs. surgery	Resectable + BRPC	SR/MA (mixed)	Quality tool	Not clear	Moderate	High–Unclear	Medium	A4	No
Lee YS (2019; *Sci Rep*) [33]	NAT vs. surgery	Resectable	SR/MA (mixed; ITT vs. PP)	MINORS	Not clear	Low	High	Low	A3	No
Unno M (2019; *Surg Today*) [34]	NAT outcomes	Mixed/unclear	SR/MA (mixed)	Quality tool	Not clear	Low	High–Unclear	Low	D2 (early/broad NAT SRMAs)	No
Dhir M (2017; *WJSO*) [35]	NAT feasibility/outcomes	Mixed/unclear	SR/MA (very broad)	Not uniform	Not clear	Low	High	Low	D2	No
Zhan HX (2017; *Cancer Med*) [36]	NAT prospective studies	Mixed/unclear	SR/MA (prospective)	Quality tool	Not clear	Low–Moderate	High–Unclear	Low	D2	No
D’Angelo F (2017; *Med Oncol*) [37]	NAT (evidence review + MA)	Mixed/unclear	SR/MA (mixed)	Quality tool	Not clear	Low	High–Unclear	Low	D2	No
Andriulli A (2012; *Ann Surg Oncol*) [38]	Preop gemcitabine (localized)	Mixed/unclear	SR/MA (prospective)	Quality tool (older)	Not clear	Low	High	Low	D2	No
Versteijne E (2018; *BJS*) [39]	NAT vs. surgery	Resectable + BRPC	SR/MA (mixed; medians)	Quality tool	Not clear	Moderate	High–Unclear	Medium	A4	No
Gillen S (2010; *PLoS Med*) [40]	NAT outcomes (response/resection %)	Resectable + borderline/unresectable groups	SR/MA of proportions (single-arm; broad eras/designs)	No standard study-level RoB tool; quality assessed “in the style of GRADE profiler”	Funnel plots created for key outcomes (e.g., resection rate)	Critically low	High	Low	D2 (early/broad NAT SRMAs)	No
Xu (2021; Bioscience Trends) [41]	NAT vs. surgery	Resectable	SR/MA (mixed)	Quality tool	Funnle plot (low risk)	Low-moderate	High	Low	A3	No

AMSTAR-2 overall: High/Moderate/Low/Critically Low; ROBIS overall: Low/High/Unclear; Integrated UR quality grade (Liu): High (AMSTAR-2 High/Moderate and ROBIS Low), Medium (meets one), Low (meets neither); Index SRMA: recommended primary SRMA within a high-overlap cluster (others used for triangulation/sensitivity).

**Table 2:** Results of meta-analysis included in umbrella review.

Meta-Analysis	Population/Comparison	Included Studies/N	Survival Outcomes	Surgery/Pathology Outcomes	Key Notes/Safety
Gillen 2010 [40]	Pancreatic and periampullary cancers; stratified by initial resectability (Group 1 resectable) Comparison: Single-arm: preop therapy → restaging → exploration/resection	111 studies (mixed prospective/retrospective; incl. 56 phase I–II); N = 4394	Group 1 (initially resectable): median OS 23.3 mo (resected 23.2; unresected 16.2); Group 1 1 year survival 65.5%, 3 year 24.9%	Group 1: exploration 90.2%; resection 73.6%; R0 (among resected) 82.1%	Very heterogeneous regimens; non-comparative pooled proportions
Andriulli 2012 [38]	Localized pancreatic cancer (initially resectable or unresectable) Comparison: Single-arm: neoadjuvant gemcitabine ± RT then surgery	20 prospective studies; N = 707 (366 resectable; 341 unresectable)	After resection: 1 year survival 91.7% and 2 year 67.2% (initially resectable)	Initially resectable: 91% underwent surgery after restaging; 82% of explored resected; R0 89%	Grade 3–4 toxicity 31% (treatment-related)
Xu CP 2014 [25]	Resectable pancreatic cancer (incl. some periampullary in source trials) Comparison: CRT vs. non-CRT; and neoadjuvant CRT vs. adjuvant CRT	CRT vs. non-CRT: 17 studies; NCRT vs. adjuvant CRT: 3 studies; N = 3088; 189	CRT vs. non-CRT: OS HR 0.96 (95% CI 0.89–1.03); PFS HR 0.83 (0.68–1.03). NCRT vs. adjuvant CRT: OS HR 0.93 (0.69–1.25)	Not the focus of this review (survival meta-analysis; limited surgical end points reported in abstract)	Includes older CRT-era studies; mixed designs
Liu W 2016 [24]	Resectable pancreatic adenocarcinoma Comparison: NCRT → surgery vs. surgery alone	8 studies (mixed RCT/prospective/retrospective); N = 833	OS HR 0.87 (95% CI 0.75–1.00; *p* = 0.051) (borderline NS)	R0 (complete) resection OR 2.39 (1.21–4.74); in-hospital mortality OR 1.27 (0.35–4.58)	No publication bias test (<10 studies)
D’Angelo 2017 [37]	Non-metastatic pancreatic adenocarcinoma (resectable/BR/LA; prospective datasets) Comparison: Single-arm: neoadjuvant protocols with ITT reporting	12 studies (prospective/prospectively collected); N = 624	ITT median OS 16.7 mo (95% CI 15.16–18.26); resected OS 22.78 mo; unresected OS 9.89 mo	Not detailed in abstract beyond survival (protocol achievement/R0 discussed in paper)	Authors conclude evidence insufficient outside RCT context
Dhir 2017 [35]	Pancreatic adenocarcinoma across resectability strata (resectable, BR, LA)—ITT pooled rates Comparison: Single-arm: neoadjuvant therapy (CT/CRT) → surgery when feasible	96 studies; N = 5520	Median OS for resectable after NAT: 30.0 mo (resected); borderline: 27.4 mo (resected) (ITT medians reported across strata)	ITT R0 resection: resectable 63%; LA 23% (R0 among resected generally >80%)	Grade ≥ 3 toxicity pooled 36%; single-arm without comparator
Zhan 2017 [36]	Prospective studies; resectable vs. BR/LA subgroups Comparison: Single-arm: neoadjuvant therapy (mostly CT ± RT)	39 prospective studies; N = 1458	Overall median OS 16.79 mo; resected 24.24 mo; unresected 9.81 mo; resectable subgroup OS 17.76 mo	Resection rate overall 57.7% (resectable 73.0%); R0 among resected 84.2% (resectable 88.2%)	Grade 3/4 toxicity 11.3%; authors caution benefit in resectable disease not proven
Huang 2018 [26]	Resectable pancreatic adenocarcinoma Comparison: NCRT → surgery vs. surgery alone (RCTs only)	2 RCTs; N = 104	OS (months): WMD +2.65 (95% CI −0.88 to 6.19; *p* = 0.14) (NS)	R0 RR 1.18 (0.76–1.81) (NS); laparotomy RR 0.82 (0.72–0.94); adverse events RR 0.80 (0.63–1.03)	Small, early-terminated RCTs; low power
Versteijne 2018 [39]	Resectable or borderline resectable pancreatic cancer Comparison: Neoadjuvant treatment vs. upfront surgery (ITT survival)	38 studies (mixed; ITT median OS required); N = 3484	Weighted median OS (ITT): 18.8 vs. 14.8 mo; (resected: 26.1 vs. 15.0 mo)	Resection rate 66.0% vs. 81.3%; R0 among resected 86.8% vs. 66.9%; R0 (ITT) 58.0% vs. 54.9%; LN+ 43.8% vs. 64.8%	Toxicity ≥ grade III up to 64%; observational dominance
Unno 2019 [34]	Resectable or borderline resectable pancreatic cancer Comparison: Neoadjuvant vs. upfront surgery (comparative ITT)	6 comparative studies (2 RCT + 4 retrospective)	OS HR 0.66 (95% CI 0.50–0.87; *p* = 0.003)	Not reported in abstract beyond survival	Considerable heterogeneity (I^2^ = 62%)
Lee 2019 [33]	Resectable pancreatic cancer (comparative studies; ITT vs. PP analyses) Comparison: Neoadjuvant (mostly CRT) vs. upfront surgery	14 studies (RCT + non-RCT; incl. database studies); N = ≈9691 (2699 NAT; 6992 US)	PP OS HR 0.72 (0.68–0.76); ITT OS HR 0.96 (0.82–1.12) (NS); among resected: NAT vs. US + adjuvant HR 0.82 (0.71–0.93)	Resection rate OR 0.46 (0.25–0.85) (lower with NAT); R0 OR 1.53 (1.35–1.73); LN metastasis OR 0.37 (0.26–0.52)	Large observational influence; attrition higher with NAT (36.3% vs. 17.3%)
Ye 2020 [31]	Primary resectable pancreatic cancer Comparison: Neoadjuvant chemotherapy vs. upfront resection	11 studies (8 cohort, 3 RCT); N = 9773	OS HR 0.86 (0.73–1.03; *p* = 0.10) (NS); gemcitabine-based NAC subgroup HR 0.75 (0.57–0.99)	R0 OR 2.62 (1.70–4.03); LN + OR 0.34 (0.31–0.37); unresectable rate higher with NAC (OR 2.18, 1.41–3.37)	Most studies ITT; chemotherapy-only focus (no RT)
Cloyd 2020 [16]	Resectable and borderline resectable pancreatic cancer Comparison: Neoadjuvant therapy (CT ± RT) vs. upfront surgery ± adjuvant	17 studies (1 RCT; rest retrospective); N = 2286	Resectable: OS HR 0.75 (0.58–0.98); DFS HR 0.73 (0.59–0.89). Borderline: OS HR 0.48 (0.31–0.74); DFS HR 0.61 (0.44–0.86)	Resection OR 0.46 (0.32–0.66); R0 OR 1.59 (1.41–1.80); LN + OR 0.45 (0.39–0.52)	No difference in major complications OR 0.96; 90-day mortality OR 0.79
Pan 2020 [32]	Resectable vs. borderline resectable subgroups Comparison: Neoadjuvant therapy vs. surgery first	17 trials (comparative); N = 2286	BRPC: OS (ITT) HR 0.49 (0.37–0.65); OS (resected) HR 0.66 (0.51–0.85). RPC: OS (ITT) HR 1.02 (0.85–1.22); OS (resected) HR 0.75 (0.63–0.89)	BRPC: resection OR 0.69 (0.41–1.16) (NS). RPC: resection OR 0.50 (0.25–0.99). (R0 generally higher with NAT; details in paper)	Stratified conclusions: benefit concentrated in BRPC, not clear in pure resectable ITT
Xu (BioScience Trends) 2021 [41]	Resectable pancreatic cancer Comparison: Neoadjuvant therapy vs. upfront surgery	11 studies (comparative); N = 9656	OS HR 0.74 (0.70–0.78) (reported); authors note no significant OS increase despite HR < 1	R0 OR 1.59 (1.41–1.80); T stage < 2 OR 2.22 (1.62–3.06); LN + OR 0.35 (0.27–0.46)	Interpretation inconsistencies; largely non-RCT evidence
Janssen 2021 [21]	Resectable + borderline resectable PDAC treated with neoadjuvant FOLFIRINOX Comparison: FOLFIRINOX alone vs. FOLFIRINOX + radiotherapy	15 studies (cohort; no RCTs); N = 512	Median OS: 21.6 mo (with RT) vs. 22.4 mo (no RT) (*p* = 0.65)	Resection rate 71.9% vs. 63.1% (*p* = 0.43); R0 88.0% vs. 97.6% (*p* = 0.045)	RT associated with higher R0 but similar OS; observational data
van Dam 2022 [15]	Resectable and borderline resectable pancreatic cancer Comparison: Neoadjuvant (CT ± RT) vs. upfront surgery (RCTs only)	7 RCTs; N = 938	OS HR 0.66 (0.52–0.85) overall; resectable subgroup HR 0.77 (0.53–1.12) (NS); borderline subgroup HR 0.61 (0.44–0.85)	Resection RR 0.83 (0.77–0.89); R0 among resected RR 1.38 (1.25–1.52); R0 (ITT) RR 1.20 (1.10–1.32); N0 RR 1.68 (1.43–1.97)	Grade 3–4 AEs higher with neoadjuvant RR 1.38; postop morbidity similar
Luo 2022 [23]	Resectable pancreatic cancer Comparison: Neoadjuvant chemoradiotherapy vs. upfront surgery	5 studies (comparative); N = 437	2 year OS OR 1.60 (1.10–2.33) (better with NCRT)	R0 OR 3.38 (2.14–5.34); serious AEs OR 0.56 (0.31–1.00) (borderline)	Small evidence base; effect measures sometimes inconsistently reported
De Simoni 2022 [27]	Resectable + borderline resectable pancreatic cancer Comparison: Total neoadjuvant therapy (systemic CT + CRT) vs. neoadjuvant systemic therapy without CRT	8 studies (comparative); N = 1080	2 year OS OR 1.64 (1.20–2.24) in favor of TNT	Radicality (R0) OR 2.03 (1.40–2.96); LN status improved OR 1.77 (1.28–2.43)	Heterogeneous TNT definitions; mostly non-randomized
Ghanem 2022 [13]	Resectable pancreatic adenocarcinoma Comparison: Neoadjuvant chemotherapy ± radiotherapy vs. upfront surgery (RCTs)	8 RCTs; N = 982	OS HR 0.75 (0.58–0.98); DFS HR 0.73 (0.59–0.89)	Resection RR 0.92 (0.86–0.97); R0 RR 1.31 (1.16–1.47)	Supports survival benefit but with lower resection rate
Uson Junior 2023 [31]	Resectable pancreatic cancer Comparison: Neoadjuvant therapy vs. upfront surgery (RCTs)	6 RCTs; N = 805	OS HR 0.76 (0.52–1.11) (NS); DFS HR 0.71 (0.46–1.09) (NS)	R0 higher by ~20%: RR 1.20 (1.04–1.37) (measure labeled inconsistently in abstract)	Authors conclude improved margin status but no proven survival benefit
Hajibandeh 2023 [19]	Resectable + borderline resectable pancreatic cancer Comparison: Neoadjuvant CRT vs. immediate surgery (RCTs)	4 RCTs; N = 400	OS mean difference +3.75 years (0.76–6.74) (overall); BRPC subgroup +6.64 years (4.77–8.51); RPC subgroup NS	R0 RR 1.55 (1.16–2.07); resection RR 0.83 (0.74–0.93)	Benefit driven by BRPC; small RCT evidence
Yang (Clin Exp Med) 2023 [28]	Resectable pancreatic cancer Comparison: Neoadjuvant therapy vs. upfront surgery	24 studies (incl. 6 RCTs); N = 3881	OS HR 0.73 (0.65–0.82); DFS HR 0.72 (0.62–0.84); RCT subgroup OS HR 0.72 (0.58–0.90)	Resection OR 0.43 (0.33–0.55); R0 OR 2.05 (1.47–2.88); LN + OR 0.38 (0.27–0.52)	Observational dominance; shows trade-off of attrition vs. improved margins
Roesel 2023 [30]	Primarily resectable pancreatic adenocarcinoma Comparison: Neoadjuvant therapy (CT or CRT) vs. upfront surgery	15 studies (mixed RCT/retrospective)	OS HR 0.80 (0.72–0.90); DFS HR 0.66 (0.56–0.79)	R0 OR 1.70 (1.23–2.36); LN + OR 0.45 (0.32–0.63)	High heterogeneity noted by authors; total N not in abstract
Yang (Updates Surg) 2024 [29]	Resectable + borderline resectable pancreatic cancer Comparison: Neoadjuvant therapy vs. upfront surgery (with/without adjuvant)	50 studies	OS HR 0.74 (*p* < 0.001); RFS HR 0.75 (*p* = 0.006); NAT vs. adjuvant-only HR 0.87 (*p* = 0.019)	R0 OR 1.90 (*p* < 0.001); LN metastasis OR 0.36; perineural invasion OR 0.42; resection rate OR 0.42 (lower with NAT)	Large, heterogeneous evidence base (incl. databases)
Bao 2024 [22]	Resectable PDAC receiving neoadjuvant treatment Comparison: Neoadjuvant CRT vs. chemotherapy-only (pathologic response focus)	11 studies; N = 4129 (1830 NAC; 2299 NCRT)	3 year OS OR 1.07 (0.84–1.36) (NS)	pCR OR 3.58 (2.10–6.08); R0 OR 1.49 (1.21–1.84)	Focus on pathologic response;
Aliseda 2024 [12]	Resectable pancreatic cancer Comparison: Neoadjuvant therapy vs. upfront surgery (RCTs; individual patient data reconstructed)	7 RCTs (reconstructed IPD); N = 1191	OS HR 0.88 (0.76–1.01) (*p* = 0.07) (NS); Disease-free time HR 0.84 (0.73–0.96)	R0 RR 1.19 (1.07–1.33); N0 RR 1.52 (1.25–1.84); resection RR 0.89 (0.82–0.98)	Higher grade 3–4 AEs RR 1.58 (1.02–2.45)
Tanadi 2025 [10]	Resectable pancreatic cancer Comparison: Neoadjuvant therapy vs. upfront surgery (RCTs)	7 RCTs; N = 571	OS HR 0.92 (0.72–1.18) (NS); DFS HR 0.98 (0.80–1.20) (NS)	R0 RR 1.31 (1.11–1.55) (higher with NAT)	Authors conclude R0 improved without OS/DFS benefit
Tan HL 2025 [6]	Resectable pancreatic cancer Comparison: Neoadjuvant therapy vs. upfront surgery (RCTs)	9 RCTs; N = 1131	OS HR 0.85 (0.68–1.05) (NS); EFS HR 0.77 (0.65–0.90)	R0 RR 1.13 (1.00–1.27); pN0 RR 1.73 (1.31–2.28); exploration RR 0.90 (0.87–0.94)	Largest RCT-only set; demonstrates EFS improvement and path downstaging
Chan 2025 [9]	Resectable pancreatic cancer Comparison: Neoadjuvant therapy vs. upfront surgery (RCTs)	8 RCTs; N = 982	OS HR 0.81 (*p* = 0.06) (NS); DFS HR 0.66 (*p* = 0.001)	R0 RR 1.20; pN0 RR 1.68; resection RR 0.95; exploration RR 0.84	Directionally favors NAT for DFS and margins; OS borderline
Annesi 2025 [18]	Resectable and borderline resectable pancreatic cancer Comparison: Neoadjuvant therapy vs. upfront surgery (RCTs)	10 RCTs; N = 1340	OS HR 0.78 (0.61–0.99) overall; resectable-only subgroup HR 0.86 (0.59–1.26) (NS)	Not detailed in abstract	Benefit largely from BRPC; RCT-only
Wu 2025 [20]	Resectable and borderline resectable PDAC receiving neoadjuvant chemotherapy Comparison: NAC + chemoradiation vs. NAC alone	8 studies; N = 1019	Median OS 25.55 mo (NAC + CRT) vs. 17.55 mo (NAC alone) (*p* < 0.001)	R0 and N0 rates significantly higher with NAC + CRT (pooled) (exact effect measures not in abstract)	Observational + RCT mix; comparison is within neoadjuvant strategies
Zuo 2025 [11]	Resectable pancreatic cancer Comparison: Neoadjuvant therapy vs. upfront surgery (RCTs)	6 RCTs; N = 670	OS HR 0.91 (0.74–1.11) (NS); DFS HR 0.82 (0.56–1.20) (NS)	R0 RR 1.41 (1.14–1.75); major complications RR 0.82 (0.68–0.98)	RCT-only; margins better, no survival benefit
Dickerson 2025 [17]	Resectable + borderline resectable PDAC (subgroups analyzed) Comparison: Neoadjuvant therapy vs. upfront surgery (RCTs)	9 RCTs; N = 1194	OS HR 0.73 (0.55–0.98); PFS HR 0.80 (0.65–0.99)	Resection RR 0.94 (0.89–0.99); R0 RR 1.35 (1.16–1.57); N0 RR 2.03 (1.50–2.74)	Grade ≥ 3 AEs higher with NAT (reported); OS benefit strongest in BRPC subgroup

**Table 3:** Summarizes the main results of the umbrella review.

Index/Meta-Analysis	PICO/Population	Evidence Base	Overall Survival	DFS/EFS	Resection/Exploration	R0 Resection	N0/pN0	Safety/Other Key Signals
Tan HL 2025 (Surgery) [6]	NAT vs. upfront surgery in resectable PDAC (RCTs)	9 RCTs; N = 1131 (604 NAT; 527 upfront)	HR 0.85 (95% CI 0.68–1.05), NS; TSA: OS inconclusive	EFS HR 0.77 (0.65–0.90), significant; TSA: EFS definitive	Resection 76.0% vs. 82.2% (NS); exploration RR 0.90 (0.87–0.94)	RR 1.13 (1.00–1.27), borderline; TSA: R0 inconclusive	pN0 RR 1.73 (1.31–2.28)	“Other outcomes comparable”; bias assessment reported low overall
Chan AHY 2025 (Ann Surg Oncol) [9]	NAT vs. upfront surgery in resectable PDAC (RCTs)	8 RCTs (OS), 7 RCTs (DFS); resection outcomes 8 RCTs	HR 0.81 (0.64–1.01), *p* = 0.06 (NS)	DFS HR 0.66 (0.48–0.92), *p* = 0.01	Resection RR 0.95 (0.89–1.03), *p* = 0.21	RR 1.20 (1.01–1.43), *p* = 0.03	pN0 RR 1.68 (1.28–2.20), *p* < 0.001	GRADE/RoB reported; funnel/Egger reported (caution: few studies)
van Dam JL 2022 (Eur J Cancer) [15]	NAT vs. upfront surgery in resectable + borderline resectable PDAC (RCTs)	7 RCTs; N = 938	Overall: HR 0.66 (0.52–0.85), *p* = 0.001; resectable-only HR 0.77 (0.53–1.12), NS; BRPC HR 0.61 (0.44–0.85)	(Not primary endpoint; Table includes other outcomes)	Resection RR 0.94 (0.89–1.01), *p* = 0.08	RR 1.47 (1.17–1.84), *p* < 0.001	N0 RR 2.15 (1.69–2.72), *p* < 0.001	Major surgical complications RR 0.60 (0.34–1.05), *p* = 0.08; serious AEs descriptively higher with NAT
Hajibandeh 2023 (AHPBS) [19]	Neoadjuvant CRT vs. immediate surgery in RPC/BRPC (RCTs; TSA)	4 RCTs; N = 400	Overall: MD 3.75 (0.93–6.56), *p* = 0.009; RPC MD 0.94 (−2.27–4.15), NS; BRPC MD 6.64 (2.13–11.14), *p* = 0.004	—	Overall: resection RR 0.83 (0.72–0.95), *p* = 0.008; RPC RR 0.84 (0.71–0.99), *p* = 0.04; BRPC RR 0.80 (0.63–1.02), NS	Overall: RR 1.55 (1.15–2.09), *p* = 0.004; RPC RR 1.18 (0.95–1.46), NS; BRPC RR 3.72 (1.53–9.07), *p* = 0.004	—	TSA: information size not reached for some endpoints; subgroup pattern suggests benefit mainly in BRPC
Bao 2024 (Pancreatology) [22]	NACRT vs. NAC (after NAT; mixed stages incl resectable/BR/LA; pathologic-response focus)	11 studies; N = 4129	3-year OS OR 1.07 (0.84–1.36), NS	—	—	R0 OR 1.49 (1.17–1.90), *p* = 0.001	—	pCR OR 3.58 (2.47–5.18), *p* < 0.00001 (higher with CRT)
Gillen 2010 (PLoS Med) [40]	Single-arm neoadjuvant therapy: response/resection percentages and survival estimates (resectable vs. non-resectable at baseline)	111 studies; N = 4394	Median survival after resection: 23.3 mo (resectable group) vs. 20.5 mo (initially non-resectable group)	—	Resectability after NAT: 73.6% (initially resectable) vs. 33.2% (initially non-resectable)	R0 among resected: 82.1% (resectable) vs. 79.2% (non-resectable)	—	Grade 3/4 toxicity 29.4% overall; heterogeneity high; non-comparative (contextual)

## Data Availability

No new data were created because this was a review of the literature data.

## Supplementary Materials

The following supporting information can be downloaded at: https://www.mdpi.com/article/10.3390/cancers18091344/s1, Table S1: Summary of pooled estimates across systematic reviews/meta-analyses by outcome; Table S2: Resectability definitions across key index RCTs; Table S3: Resectability definitions across key index RCTs; Table S4: Evidence-based clinical decision framework: NAT vs. upfront surgery in operable PDAC.
