# Supplementary material for: Upfront Chemotherapy Versus Immediate Surgery for Operable Pancreatic Cancer: An Umbrella Review of Meta-Analyses
Source: Cancers (Basel). 2026 Apr 23;18(9):1344. doi: 10.3390/cancers18091344 (PMC13163042; doi:10.3390/cancers18091344)
Supplement: Supplementary file 1 [file cancers-18-01344-s001.zip › cancers-4242582-supplementary.pdf]

Supplementary Table S1. Summary of Pooled Estimates Across Systematic Reviews/Meta-Analyses by Outcome

Neoadjuvant Therapy vs Upfront Surgery for Operable Pancreatic Cancer — Umbrella Review of 34 SRMAs (2010–2025)

Outcome	SRMA	Population	Design	Measure	Estimate (95% CI)
**Overall Survival (OS)**	**Tan 2025**	R	RCT	**HR**	0.85 (0.68–1.05)
	**Chan 2025**	R	RCT	**HR**	0.81 (0.64–1.01)
	**Annesi 2025**	R	RCT	**HR**	0.75 (0.55–1.02)
	**Aliseda 2024**	R	RCT	**HR**	0.82 (0.65–1.03)
	**Roesel 2023**	R	Mixed	**OR**	**1.70 (1.23–2.36)**
	**Uson Junior 2023**	R	RCT	**HR**	0.79 (0.59–1.07)
	**Yang B 2023**	R+BR	Mixed	**HR**	**0.80 (0.70–0.92)**
	**Ghanem 2022**	R+BR	RCT	**HR**	**0.66 (0.52–0.85)**
	**van Dam 2022**	R+BR	RCT	**HR**	**0.75 (0.58–0.98)**
	**Xu 2022**	R	Mixed	**HR**	0.86 (0.73–1.03)
	**Cloyd 2020**	R+BR	RCT	**HR**	**0.73 (0.61–0.86)**
	**Ye 2020**	R	Mixed	**HR**	0.86 (0.73–1.03)
	**Lee 2019**	R+BR	Mixed	**HR**	**0.80 (0.70–0.92)**
	**Lee 2019 (ITT)**	R+BR	Mixed	**HR**	0.96 (0.82–1.12)
	**Unno 2019**	R+BR	Mixed	**HR**	**0.82 (0.71–0.93)**
	**Liu 2016**	R	Mixed	**HR**	0.85 (0.58–1.25)
	**Versteijne 2018**	R+BR	Mixed	**HR**	0.87 (0.74–1.02)
	**Tanadi 2025**	R	RCT	**HR**	0.92 (0.72–1.18)
	**Dickerson 2025**	R+BR	RCT	**HR**	**0.72 (0.55–0.94)**
	**Zuo 2025**	R	RCT	**HR**	0.78 (0.56–1.10)
	**Wu 2025**	R+BR	RCT	**HR**	**0.84 (0.71–0.99)**
	**Luo 2022**	R+BR	Mixed	**HR**	0.87 (0.74–1.02)
	**Hajibandeh 2023**	R+BR	RCT	**HR**	**0.77 (0.61–0.98)**
**Disease-Free / Event-Free Survival (DFS/EFS)**	**Tan 2025**	R	RCT	**HR**	**0.77 (0.65–0.90)**
	**Chan 2025**	R	RCT	**HR**	**0.66 (0.48–0.92)**
	**Ghanem 2022**	R+BR	RCT	**HR**	**0.73 (0.59–0.89)**
	**Tanadi 2025**	R	RCT	**HR**	0.98 (0.80–1.20)
	**Dickerson 2025**	R+BR	RCT	**HR**	0.69 (0.47–1.02)
	**Annesi 2025**	R+BR	RCT	**RR**	**0.74 (0.63–0.88)**
**R0 (Margin-Negative) Resection Rate**	**Tan 2025**	R	RCT	**RR**	1.13 (1.00–1.27)
	**Chan 2025**	R	RCT	**RR**	1.18 (0.95–1.46)
	**Cloyd 2020**	R+BR	RCT	**RR**	**1.51 (1.18–1.93)**
	**Ghanem 2022**	R+BR	RCT	**RR**	**1.31 (1.13–1.52)**
	**Hajibandeh 2023**	R+BR	RCT	**RR**	**1.55 (1.15–2.09)**
	**Hajibandeh 2023 (BRPC)**	BR	RCT	**RR**	**3.72 (1.53–9.07)**
	**Roesel 2023**	R	Mixed	**OR**	**1.70 (1.23–2.36)**
	**Tanadi 2025**	R	RCT	**RR**	**1.31 (1.11–1.55)**
	**Liu 2016**	R	Mixed	**OR**	**2.78 (1.12–6.92)**
	**Ye 2020**	R	Mixed	**OR**	**2.18 (1.41–3.37)**
	**Yang S 2024**	R+BR	RCT	**OR**	**3.02 (1.88–4.85)**
	**Annesi 2025**	R	RCT	**RR**	**1.10 (1.02–1.18)**
	**Dickerson 2025**	R+BR	RCT	**RR**	**1.24 (1.05–1.47)**
	**Wu 2025**	R+BR	RCT	**RR**	**1.55 (1.15–2.09)**
**Node-Negative (pN0) Rate**	**Tan 2025**	R	RCT	**RR**	**1.73 (1.31–2.28)**
	**Chan 2025**	R	RCT	**HR**	**1.71 (1.25–2.35)**
	**Cloyd 2020**	R+BR	RCT	**RR**	**2.07 (1.47–2.91)**
	**Roesel 2023**	R	Mixed	**OR**	**0.45 (0.32–0.63)**
	**Tanadi 2025**	R	RCT	**HR**	**1.94 (1.23–3.06)**
	**Yang S 2024**	R+BR	Mixed	**OR**	**0.36 (0.22–0.59)**
	**Dickerson 2025**	R+BR	RCT	**RR**	**1.65 (1.20–2.27)**
**Overall Resection Rate**	**Tan 2025**	R	RCT	**RR**	**0.90 (0.87–0.94)**
	**Chan 2025**	R	RCT	**RR**	0.95 (0.89–1.03)
	**Ghanem 2022**	R+BR	RCT	**RR**	0.92 (0.84–1.01)
	**Hajibandeh 2023**	R+BR	RCT	**RR**	**0.83 (0.72–0.95)**
	**Cloyd 2020**	R+BR	RCT	**RR**	0.93 (0.82–1.04)
	**Lee 2019**	R+BR	Mixed	**OR**	**0.46 (0.25–0.85)**
	**Annesi 2025**	R	RCT	**RR**	**1.10 (1.02–1.18)**
	**Dickerson 2025**	R+BR	RCT	**RR**	0.94 (0.89–1.01)
**pCR & Outcomes (CRT vs Chemo)**	**Bao 2024**	R+BR+LA	Mixed	**OR**	**3.58 (2.42–5.28)**
	**Bao 2024 (R0)**	R+BR+LA	Mixed	**OR**	**1.49 (1.15–1.94)**
	**Bao 2024 (3y-OS)**	R+BR+LA	Mixed	**OR**	1.07 (0.84–1.36)

Abbreviations: SRMA = systematic review/meta-analysis; R = resectable; BR = borderline resectable; LA = locally advanced; RCT = randomized controlled trial; HR = hazard ratio; RR = risk ratio; OR = odds ratio; CI = confidence interval; I^2 = heterogeneity; OS = overall survival; DFS = disease-free survival; EFS = event-free survival; pN0 = pathological node-negative; pCR = pathological complete response; CRT = chemoradiotherapy; NAT = neoadjuvant therapy; UFS = upfront surgery; TSA = trial sequential analysis; ITT = intention-to-treat; PP = per-protocol.

✦ = statistically significant (95% CI excludes null value). Estimates with HR/RR <1 for OS/DFS/EFS favor NAT; RR/OR >1 for R0/pN0 favor NAT; RR <1 for resection rate indicates lower resection with NAT (attrition).

Supplementary Table S2. Resectability Definitions Across Key Index RCTs

Trial / RCT	Population	Resectability Criteria Used	Key Notes
Palmer 2007 (ESPAC pilot)	R	Surgeon assessment; no vascular involvement	No formal NCCN/AHPBA criteria; institutional
Golcher 2015	R	No portal vein/SMA involvement on CT	Predates NCCN 2016; no BR category
Casadei 2015	R	CT-based; no arterial/venous involvement	No standardized BR definition
Brunner 2015	R	No vascular contact; resectable per CT	Single-center criteria
Jang 2018	R + BR	NCCN 2014 criteria	First Korean RCT to include BR
PREOPANC-1 (Versteijne 2020)	R + BR	DPCG/NCCN 2013; ≤90° SMA, ≤270° PV/SMV	Dutch criteria; ~50% BR enrolled
Prep-02/JSAP-05 (Unno 2019)	R	JPS 2009 criteria; no vascular invasion	Japanese criteria; stricter than NCCN R
NEONAX (Schwarz 2022)	R	NCCN 2015; no arterial contact, ≤180° PV	Strictly resectable; excluded BR
A021806 (Sohal 2023)	R	NCCN 2019; no arterial contact, PV/SMV patent	Strictly resectable; US Alliance trial
PANACHE-01 (Ahmad 2023)	R + BR	NCCN 2017 criteria	French multicenter
Seufferlein 2023	R	NCCN criteria; resectable only	German phase II
PREOPANC-2 (Janssen 2025)	R + BR	DPCG/NCCN 2016; same as PREOPANC-1	~50% BR; Dutch criteria
Labori 2024 (NorPACT-1)	R	NCCN 2014; no arterial contact	Scandinavian; strictly resectable
CASSANDRA (Reni 2026)	R + BR	NCCN 2017; stages I–III	Italian; resectability not independent prognostic factor

Abbreviations: R = resectable; BR = borderline resectable; NCCN = National Comprehensive Cancer Network; DPCG = Dutch Pancreatic Cancer Group; AHPBA = Americas Hepato-Pancreato-Biliary Association; JPS = Japan Pancreas Society; SMA = superior mesenteric artery; PV = portal vein; SMV = superior mesenteric vein; CT = computed tomography.

Supplementary Table S3. Resectability Definitions Across Key Index RCTs

Trial / RCT	Population	Resectability Criteria Used	Key Notes
Palmer 2007 (ESPAC pilot)	R	Surgeon assessment; no vascular involvement	No formal NCCN/AHPBA criteria; institutional
Golcher 2015	R	No portal vein/SMA involvement on CT	Predates NCCN 2016; no BR category
Casadei 2015	R	CT-based; no arterial/venous involvement	No standardized BR definition
Brunner 2015	R	No vascular contact; resectable per CT	Single-center criteria
Jang 2018	R + BR	NCCN 2014 criteria	First Korean RCT to include BR
PREOPANC-1 (Versteijne 2020)	R + BR	DPCG/NCCN 2013; ≤90° SMA, ≤270° PV/SMV	Dutch criteria; ~50% BR enrolled
Prep-02/JSAP-05 (Unno 2019)	R	JPS 2009 criteria; no vascular invasion	Japanese criteria; stricter than NCCN R
NEONAX (Schwarz 2022)	R	NCCN 2015; no arterial contact, ≤180° PV	Strictly resectable; excluded BR
A021806 (Sohal 2023)	R	NCCN 2019; no arterial contact, PV/SMV patent	Strictly resectable; US Alliance trial
PANACHE-01 (Ahmad 2023)	R + BR	NCCN 2017 criteria	French multicenter
Seufferlein 2023	R	NCCN criteria; resectable only	German phase II
PREOPANC-2 (Janssen 2025)	R + BR	DPCG/NCCN 2016; same as PREOPANC-1	~50% BR; Dutch criteria
Labori 2024 (NorPACT-1)	R	NCCN 2014; no arterial contact	Scandinavian; strictly resectable
CASSANDRA (Reni 2026)	R + BR	NCCN 2017; stages I–III	Italian; resectability not independent prognostic

			factor

Abbreviations: R = resectable; BR = borderline resectable; NCCN = National Comprehensive Cancer Network; DPCG = Dutch Pancreatic Cancer Group; AHPBA = Americas Hepato-Pancreato-Biliary Association; JPS = Japan Pancreas Society; SMA = superior mesenteric artery; PV = portal vein; SMV = superior mesenteric vein; CT = computed tomography.

Supplementary Table S4. Evidence-Based Clinical Decision Framework: NAT vs Upfront Surgery in Operable PDAC

Clinical Scenario	Recommended Strategy	Supporting Evidence & Rationale
**STRONG SUPPORT FOR NEOADJUVANT THERAPY**		
Borderline resectable PDAC (NCCN-defined vascular involvement)	**NAT preferred<br>(Level of evidence: High)**	• Consistent OS benefit in RCT-only SRMAs (pooled HR 0.66, 95% CI 0.52–0.85)<br>• Significant improvements in R0 resection (RR up to 3.72 in BRPC subgroups) and pN0 rates<br>• Subgroup analyses across multiple SRMAs confirm survival advantage driven primarily by BR tumors<br>• NCCN, ASCO, and ESMO guidelines endorse NAT as preferred in this setting<br>• Biological rationale: higher occult systemic disease burden; greater margin-positive risk mitigated by preoperative therapy
Biologically high-risk resectable PDAC:<br>• CA 19-9 >500 U/mL<br>• Large primary tumor (>4 cm)<br>• Suspicious/borderline lymphadenopathy<br>• Poor differentiation on biopsy<br>• Significant weight loss / declining PS	**NAT reasonable<br>(Level of evidence: Moderate;<br>individualized decision)**	• In resectable-only RCT syntheses, EFS is significantly improved (HR 0.77, 95% CI 0.65–0.90; TSA conclusive)<br>• pN0 rates substantially higher with NAT (RR 1.73, 95% CI 1.31–2.28)<br>• NAT serves as biological selection: patients who progress during NAT likely had occult systemic disease and may not benefit from surgery<br>• High baseline CA 19-9, tumor size, and nodal suspicion are validated adverse prognostic factors that increase the probability of early systemic relapse<br>• Shared decision-making recommended; balance disease-control gains vs attrition risk
**APPROPRIATE FOR UPFRONT SURGERY**		
Strictly resectable PDAC with favorable biology:<br>• Normal/low CA 19-9<br>• Small tumor (<2–3 cm)<br>• No suspicious lymph nodes<br>• Good performance status (ECOG 0–1)<br>• No radiologic high-risk features	**Upfront surgery + adjuvant therapy<br>(Level of evidence: High)**	• No definitive OS advantage for NAT in strictly resectable disease (HR 0.85, 95% CI 0.68–1.05; TSA inconclusive)<br>• Upfront surgery avoids treatment-related attrition (resection rate RR 0.83–0.95 with NAT in some analyses)<br>• Adjuvant modified FOLFIRINOX (PRODIGE-24) or gemcitabine/capecitabine (ESPAC-4) are established standards with proven OS benefit<br>• Low biological risk patients have the most to lose from NAT-related delays and the least to gain from preoperative selection
Patients with significant comorbidity, frailty, or high surgical risk where NAT may cause clinical deterioration	**Upfront surgery if technically feasible; careful MDT discussion**	• NAT-associated attrition is a documented concern (resection rate RR 0.83; p = 0.008 in CRT analyses)<br>• Patients with borderline performance status may not tolerate NAT and risk losing the window for curative resection<br>• Institutional experience and supportive care capacity should be considered
**AREAS OF UNCERTAINTY / EVOLVING EVIDENCE**		
Role of modern multi-agent NAT (FOLFIRINOX, PAXG) in strictly resectable disease	**Awaiting mature RCT data<br>(CASSANDRA OS pending;<br>future trials needed)**	• Most RCT evidence informing current SRMAs used gemcitabine-based regimens<br>• CASSANDRA trial: PAXG vs mFOLFIRINOX showed EFS benefit (HR 0.63); OS data immature<br>• Unknown whether more potent systemic therapy would shift OS benefit in resectable disease<br>• Recommend enrollment in ongoing/future trials where available
Optimal NAT regimen and role of radiotherapy	**No definitive evidence for CRT over chemotherapy alone for survival**	• CRT improves pCR (OR 3.58) and R0 (OR 1.49) vs chemotherapy alone<br>• No 3-year OS difference (OR 1.07, 95% CI 0.84–1.36)<br>• Radiotherapy may be selectively beneficial for persistent vascular interface or local control priorities

Abbreviations: NAT = neoadjuvant therapy; PDAC = pancreatic ductal adenocarcinoma; BR = borderline resectable; NCCN = National Comprehensive Cancer Network; ASCO = American Society of Clinical Oncology; ESMO = European Society of Medical Oncology; CA 19-9 = carbohydrate antigen 19-9; PS = performance status; ECOG = Eastern Cooperative Oncology Group; MDT = multidisciplinary team; CRT = chemoradiotherapy; pCR = pathological complete response; TSA = trial sequential analysis; EFS = event-free survival; OS = overall survival.
